# Cancer-related cognitive impairment and wellbeing in patients with newly diagnosed aggressive lymphoma compared to population norms and healthy controls: an exploratory study

**DOI:** 10.1007/s00520-024-08441-2

**Published:** 2024-03-21

**Authors:** Priscilla Gates, Haryana. M. Dhillon, Mei Krishnasamy, Carlene Wilson, Karla Gough

**Affiliations:** 1 Cognitive Neuroscience Lab, School of Psychology, Deakin University, Burwood, Victoria Australia; 2 Department of Clinical Haematology, Austin Health, Melbourne, Victoria Australia; 3 Department of Nursing, Faculty of Medicine, Dentistry & Health Sciences, The University of Melbourne, Melbourne, Victoria Australia; 4 Health Services Research, Peter MacCallum Cancer Centre, Melbourne, Victoria Australia; 5 Faculty of Science, School of Psychology, Centre for Medical Psychology & Evidence-Based Decision-Making, The University of Sydney, Sydney, NSW Australia; 6 Olivia Newton-John Cancer Wellness and Research Centre, Austin Health, Melbourne, Victoria Australia; 7 School of Psychology and Public Health, LaTrobe University, Melbourne, Victoria Australia; 8 Faculty of Medicine, Dentistry & Health Sciences, The University of Melbourne, Melbourne, Victoria Australia

**Keywords:** Aggressive lymphoma, Cancer-related cognitive impairment, Exploratory study

## Abstract

**Purpose:**

There has been little dedicated research on cancer-related cognitive impairment in patients with aggressive lymphoma. We describe and compare patients’ cognitive function with that of healthy controls and patients’ wellbeing and distress with general population values. We also explore associations between patients’ neuropsychological test performance and self-reported cognitive function and distress.

**Methods:**

Secondary analysis of data from a feasibility study of 30 patients with newly diagnosed aggressive lymphoma and 72 healthy controls. Patients completed neuropsychological tests and self-report measures before and 6–8 weeks after chemotherapy. Healthy controls completed neuropsychological tests and the FACT-Cog at enrolment and 6 months later. Mixed models were used to analyze neuropsychological test and FACT-Cog scores. One-sample *t*-tests were used to compare patients’ self-reported wellbeing and distress with population norms. Associations were explored with Kendall’s Tau *b*.

**Results:**

Patients and healthy controls were well matched on socio-demographics. Differences between neuropsychological test scores were mostly large-sized; on average, patients’ scores on measures of information processing speed, executive function, and learning and memory were worse both before and after chemotherapy (all *p* ≤ 0.003). The same pattern was observed for impact of perceived cognitive impairment on quality-of-life (both *p* < 0.001). Patients’ physical and emotional wellbeing scores were lower than population norms both before and after chemotherapy (all *p* ≤ 0.018). Associations between neuropsychological performance and other measures were mostly trivial (all *p* > 0.10).

**Conclusion:**

For many patients with aggressive lymphoma, impaired neuropsychological test performance and impact of perceived impairments on quality-of-life precede chemotherapy and are sustained after chemotherapy. Findings support the need for large-scale longitudinal studies with this population to better understand targets for interventions to address cognitive impairments.

## Introduction

Cancer-related cognitive impairment (CRCI) is a highly distressing and disabling symptom commonly reported by patients across cancer types [1, 2]. The incidence of CRCI varies, but studies in patients diagnosed with solid tumours suggest up to 70% receiving anti-cancer treatment report some degree of cognitive impairment [1, 2]. For some cognitive impairment may be transient, but for a subgroup, these symptoms can be long-standing and have a major impact on quality of life (QoL) and activities of daily life [3, 4]. While persistent changes in cognitive function are reported among lymphoma survivors, [5, 6] most studies have focused on women with breast cancer, alongside a smattering of studies assessing other solid tumour groups [4, 7]. In studies focused on haematological malignancies, [8–11] few have included people with aggressive lymphoma.

Cognitive functioning in people with cancer can be assessed using two main approaches: i) self-reported or subjective assessment and ii) objective assessment using neuropsychological tests. Both provide important data for research and clinical practice. However, evidence suggests, at best, a weak association between these two approaches to assessment. Many individuals who self-report cognitive difficulties score within the normal range on neuropsychological tests [12, 13]. To explore associations between self-report and objective assessments of cognitive function Bray et al. [1] conducted a systematic review evaluating self-reported cognitive functioning and its associations with neuropsychological tests and patient-reported outcomes in adult cancer patients who had received chemotherapy for a non-haematological cancer. They found widespread heterogeneity in the assessment of self-reported cognitive symptoms and consistently absent or weak association with neuropsychological test scores.

We recently published our feasibility findings on the longitudinal assessment of cognition in 30 people with aggressive lymphoma undergoing standard treatment with curative intent [14]. Here, we describe and compare patients’ neuropsychological test performance and self-reported cognitive function and with that of healthy controls, who closely matched the characteristics of our sample and completed the same assessments twice, and their self-reported health-related quality of life and emotional distress with population norms. We also explore associations between patients’ neuropsychological test performance and their self-reported cognitive function and emotional distress.

## Methods

### Study design and participants

A secondary analysis of data from a longitudinal feasibility study in patients with newly diagnosed aggressive lymphoma undergoing standard chemotherapy [14] and data from a longitudinal cohort study examining cognitive function in people with colorectal cancer and healthy controls [7]. A detailed description of participants, procedures, and assessments can be found in Gates et al. [15] and Vardy et al., [16] respectively.

In brief, Gates et al.’s. [14] study was conducted in a specialized haematology department in a large acute tertiary hospital. The study enrolled people aged 18 years or older with newly diagnosed aggressive lymphoma scheduled to undergo standard combination chemotherapy with curative intent; able to read and comprehend English; and with a documented Eastern Cooperative Oncology Group (ECOG) performance status < 3. Exclusion criteria included the following: lymphomatous CNS involvement, prior or planned cranial radiotherapy and a life expectancy of < 12 months, any medical condition that might compromise adherence or lead to prolonged hospitalisation, and a documented history of past or current substance abuse, or poorly controlled psychiatric illness. Vardy et al. (2015), [7] as part of their larger prospective longitudinal study, enrolled 72 healthy controls who were from Sydney, Australia, and were generally family or friends of people with cancer.

### Assessments

Only assessments included in this secondary analysis are presented here.

Patients diagnosed with aggressive lymphoma completed neuropsychological tests before and 6–8 weeks after chemotherapy. These included the trail making test (TMT) Part A, a measure of speed of information processing, and Part B, a measure of executive function [17]; Hopkins verbal learning test (HVLT-R), a measure of learning and memory [18]; WAIS-R Digit Span, a measure of attention/working memory [19]; Stroop Color and Word Test (SCWT), a measure of executive function [20]; and Controlled Oral Word Association (COWA) test, a measure of verbal fluency [21]. Patient-reported outcome measures (PROMs) were administered at the same times. These included the FACT-Cognitive Function Version 3 (FACT-Cog), a measure of perceived cognitive impairments and abilities, the impact of perceived cognitive impairments on quality of life, and comments from others [22]. Comments from others were not included in the current analysis. The Functional Assessment of Cancer Therapy-General (FACT-G), a measure of four domains of health-related quality of life including physical, social, emotional, and functional wellbeing [23], the 7-item PROMIS Emotional Distress-Anxiety 7a [24] and 8-item PROMIS Emotional Distress-Depression 8b short-forms [25], measures of anxious and depressive symptomatology, respectively. Socio-demographic and clinical information (age, sex, marital status, and years of formal education, comorbidities, [26] prior treatment for psychiatric/neurological conditions, diagnosis, and chemotherapy duration) was gathered from the institution’s electronic medical record.

Healthy controls completed the following neuropsychological tests and the FACT-Cog at study enrolment and six months later: TMT Part A and B, HVLT-R, and WAIS-R Digit Span. Socio-demographic and clinical data were self-reported by the participants.

### Statistical considerations

#### Sample size

As with all secondary analyses, sample sizes were constrained by the availability of data in existing datasets. Based on numbers of patients and healthy controls with baseline and follow-up assessments (*n* = 29 and *n* = 72, respectively) [7, 14], sensitivity power analysis indicated 80% power to detect a difference of 0.62 SD between groups on self-reported cognitive function and neuropsychological test outcomes, using a two-sided *α* = 0.05 *t*-test. The performance of the lymphoma group was well below population normative data; on average, in the feasibility study, suggesting clinically significant impairment in this group [14, 27]. Again, based on numbers of patients with baseline and follow-up assessments (*n* = 27 to 29), sensitivity power analysis indicated 80% power to detect a difference of 0.54 SD to 0.56 SD from general population norms for the FACT-G and PROMIS short-forms using a two-sided *α* = 0.05 one-sample *t*-test. The HRQOL of the lymphoma group was below population norms on average in the feasibility study, so it was reasonable to assume we would be looking for medium to large size difference [14].

#### Analysis

Analyses were performed in R (reference index version 4.2.1). Responses to study measures were scored according to author guidelines, and for scores < 20 on the HVLT-R, were substituted with 19 to optimize available data [28]. For neuropsychological tests, a global deficit score (GDS) was calculated for each patient before and after chemotherapy to provide a global measure of neuropsychological performance. These were calculated using methods described by Carey et al. [7, 29] where the GDS is computed by converting demographically converted standard scores (*T* scores) on individual neuropsychological measures to deficit scores ranging from 0 (no impairment) to 5 (severe impairment). This was based on the following test scores: TMT A and B; HVLT-R, total and delayed recall; digit span, total; SCWT, Interference; and COWA test, total letter fluency, category fluency, and total written fluency.

Descriptive statistics were used to summarize socio-demographic, clinical, and study measure data by group (patients and healthy controls). Student’s *t*-test was used to compare groups on age and years of formal education, and Fisher’s exact test was used to compare groups on sex and marital status (married/*defacto* versus not).

Linear mixed models were used to analyse FACT-Cog subscale scores and scores based on neuropsychological tests administered to patients and healthy controls (TMT Part A and B, HVLT-R, and WAIS-R digit span). Models were fit using the ‘lme4’ package [30] and included fixed effects for group (patients, 0; healthy controls, 1), time (baseline, 0; follow-up, 1) plus a group-by-time interaction and random participant effect. The ‘lmerTest’ package [31] was used to calculate least squares means for each group at each time, as well as pairwise differences with 95% confidence intervals. Cohen’s *d* was calculated to characterise the sizes of the between-groups differences at baseline and follow-up and is interpreted as follows: 0.2, small-sized; 0.5 medium-sized; and 0.8 large-sized differences. [32] Kazis effect sizes were calculated to characterise the sizes of within-group differences between baseline and follow-up scores; these are interpreted as per Cohen’s *d* [33]*.* One-sample *t-*tests were used to compare mean patient FACT-G subscale and total scores and PROMIS short-form scores to population norms before and after chemotherapy. Where possible, evidence-based guidelines were used to support the interpretation of between-groups differences on the FACT-G. Kendall’s Tau *b* correlation was used to assess the association between patients’ GDS and FACT-Cog and PROMIS-Emotional Distress short-form scores.

## Results

### Participant characteristics

**Fig. 1:**
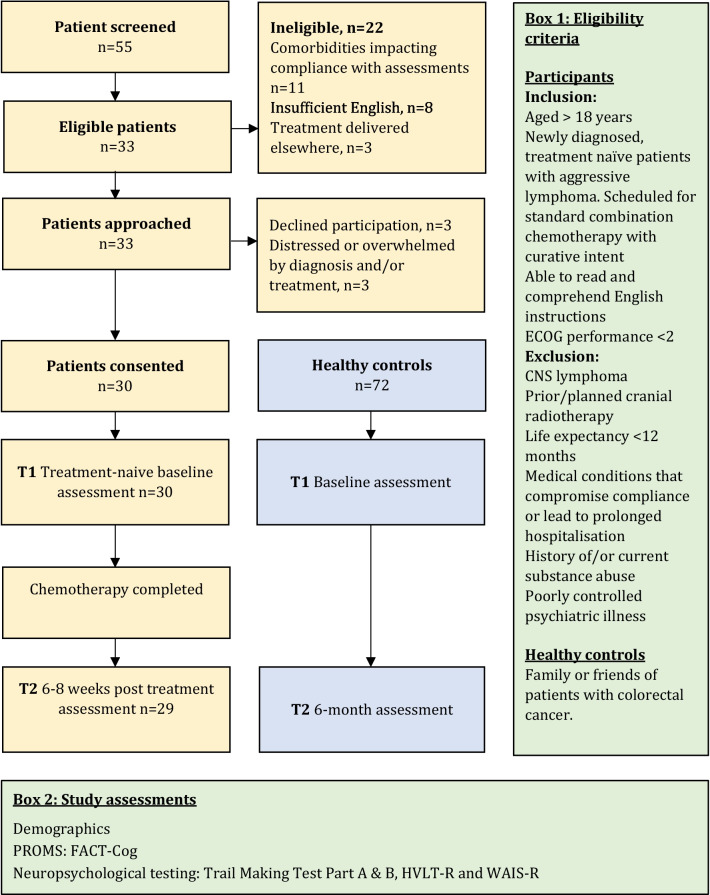
Participant flow diagram

Fifty-five patients with newly diagnosed aggressive lymphoma were screened for eligibility between 26 November 2019 and 01 September 2020. Twenty-two patients were ineligible. Reasons for ineligibility are summarized in Fig. 1.

Characteristics of patients and healthy controls are summarised in Table 1. Patients and healthy controls were similar in terms of age (*p* = 0.76), sex (*p* = 0.39), marital status (*p* = 0.82), and years of education (*p* = 0.66).

**Table 1:** Sample characteristics

Characteristics	Patients		Controls	
	*n* = 30	%	*n* = 72	%
Age at enrolment, in years				
Mean (SD)	57 (17)		56 (11)	
Median (IQR)	61 (50 to 69)		58 (46 to 63)	
Range	18 to 78		26 to 75	
Sex				
Male	16	53	31	43
Female	14	47	41	57
Marital status				
Married/de facto	21	70	47	65
Separated/divorced	2	7	8	11
Single	6	20	12	17
Widowed	1	3	5	7
Years of formal education				
Mean (SD)	13 (2)		14 (3)	
Median (IQR)	13 (12 to 14)		15 (11 to 15)	
Range	7 to 18		6 to 20	
Ever been treated/on medication for depression, anxiety, psychiatric or neurological condition^a^				
No	22	73	59	82
Yes	8	27	13	18
Diagnosis				
Anaplastic large cell	2	7		
DLBCL	20	67		
Grade 3B FL	1	3		
HL	4	13		
Mantle cell	1	3		
Peripheral T-cell	1	3		
Primary mediastinal	1	3		
Chemotherapy regime R-CHOP × 6	10	33		
R-CHOP × 4	3	10		
R-CHOP × 3	2	7		
R-CHOP × 2	1	3		
CHOP × 6	2	7		
R-CHOP & Ritux × 2	4	14		
R-CHOP & HD MTX × 2	1	3		
R-CHOP/R-DHAP × 3	1	3		
Mini R-CHOP × 6	2	7		
ABVD × 6	3	10		
Esc-BEACOPP × 4	1	3		
Length of chemotherapy treatment, in days				
Mean (SD)	102 (34)			
Median (IQR)	105 (105 to 114)			
Range	21 to 116			

^a^Ever been treated for conditions for patients; ever been on medication for healthy controls

### Neuropsychological tests

Mixed model results for neuropsychological test scores are summarised in Table 2 and least squares means are provided in Table 3.

**Table 2:** Mixed model results for self-reported cognitive function and neuropsychological performance

Measure/(sub)scale	Parameter						
	Intercept	Group		Time		Group-by-time	
	est (se)	est (se)	*p*-value	est (se)	*p*-value	est (se)	*p*-value
FACT-cognitive function							
PCI	60.8 (1.9)	− 1.8 (2.3)	0.41	− 4.0 (1.4)	0.004	4.5 (1.6)	0.008
Impact of PCI on QOL	11.3 (0.5)	3.9 (0.6)	< 0.001	0.4 (0.5)	0.47	− 0.4 (0.6)	0.50
PCA	21.7 (1.0)	− 0.8 (1.2)	0.53	− 2.4 (1.0)	0.017	2.7 (1.2)	0.024
Trail making test							
A score	45.8 (1.8)	10.0 (2.1)	< 0.001	2.9 (1.9)	0.12	0.2 (2.2)	0.91
B score	46.6 (2.1)	10.2 (2.5)	< 0.001	0.7 (2.0)	0.73	1.4 (2.3)	0.53
Hopkins verbal learning test							
Total recall	40.8 (1.8)	8.4 (2.2)	< 0.001	5.2 (1.5)	< 0.001	− 2.0 (1.8)	0.27
Delayed recall	38.1 (12.0)	11.6 (2.3)	< 0.001	3.5 (1.8)	0.046	− 2.0 (2.1)	0.34
Retention*	41.8 (1.8)	8.2 (2.2)	< 0.001	0.1 (2.3)	0.97	− 0.2 (2.7)	0.94
WAIS-R digit span							
Digit span total	48.2 (1.7)	4.4 (2.0)	0.033	1.1 (1.2)	0.34	1.6 (1.4)	0.26

**Table 3:** Least squares means table for self-reported cognitive function and neuropsychological performance in patients and healthy controls

Measure/(sub)scale	Within-groups comparisons						Between-groups comparisons					
	Patients^a^			Healthy controls^b^			Baseline			Follow-up		
	BL	FU	Chg (95% CI)	BL	FU	Chg (95% CI)	Diff (95% CI)	*p*-value	*d*	Diff (95% CI)	*p*-value	*d*
FACT-cognitive function^c^												
PCI	60.8	56.8	− 4.0 (− 6.8, − 1.3)	58.9	59.4	0.4 (− 1.3, 2.2)	1.9 (− 2.6, 6.4)	0.41	0.18	− 2.6 (− 7.1, 2.0)	0.26	0.25
Impact of PCI on QOL	11.3	11.7	0.4 (− 0.7, 1.5)	15.2	15.2	0.0 (− 0.7, 0.6)	− 3.9 (− 5.1, − 2.8)	< 0.001	1.44	− 3.5 (− 4.7, − 2.3)	< 0.001	1.27
PCA	21.7	19.3	− 2.4 (− 4.4, 0.4)	21.0	21.3	0.3 (− 1.6, 1.0)	0.8 (− 1.6, 3.2)	0.52	0.14	− 1.9 (− 4.4, 0.5)	0.11	0.35
Trail making test^d^												
A score	45.8	48.7	2.9 (− 0.7, 6.6)	55.7	58.9	3.2 (0.8, 5.5)	− 10.0 (− 14.2, − 5.8)	< 0.001	1.03	− 10.2 (− 14.4, − 6.0)	< 0.001	1.06
B score	46.6	47.3	0.7 (− 3.2, 4.6)	56.8	59.0	2.1 (− 0.3, 4.6)	− 10.2 (− 15.1, − 5.3)	< 0.001	0.90	− 11.6 (− 16.7, − 6.7)	< 0.001	1.03
Hopkins verbal learning test^d^												
Total recall	40.8	46.0	5.2 (2.2, 8.1)	49.2	52.4	3.2 (1.3, 5.1)	− 8.4 (− 12.7, − 4.1)	< 0.001	0.85	− 6.4 (− 10.7, − 2.1)	0.003	0.65
Delayed recall	38.1	41.7	3.5 (0.1, 7.0)	49.8	51.3	1.6 (− 0.7, 3.8)	− 11.6 (− 16.3, − 7.0)	< 0.001	1.09	− 9.7 (− 14.3, − 5.0)	< 0.001	0.91
Retention^e^	41.8	41.8	0.1 (− 4.4, 4.5)	50.0	49.9	0.1 (− 3.0, 2.7)	− 8.2 (− 12.6, − 3.9)	0.001	0.82	− 8.0 (− 12.4, − 3.6)	< 0.001	0.80
Digit span^d^												
Total	48.2	49.4	1.2 (− 1.2, 3.6)	52.6	55.4	2.8 (1.3, 4.3)	− 4.4 (− 8.4, − 0.4)	0.024	0.47	− 6.0 (− 10.0, − 2.0)	0.004	0.65

*BL* baseline, *FU* follow-up, *Chg* change at follow-up from baseline, *Diff* difference between patients and healthy controls, *d* Cohen’s *d*, *PCI* perceived cognitive impairment, *QOL* quality of life, *PCA* perceived cognitive abilities

^a^30 patients at baseline, 29 at follow-up (6-–8 weeks after chemotherapy)

^b^72 healthy controls at baseline and follow-up (6 months after baseline assessment)

^c^Higher scores reflect higher quality of life. Possible scores’ ranges for FACT-Cognitive Function subscales: PCI (0–72), impact of PCI on QoL (0–16), PCA (0–28)

^d^Higher scores reflect higher neuropsychological performance

^e^Three healthy controls obtained retention scores ≤ 20

The fixed effect for group was statistically significant for all subscales, whereas the group-by-time interaction was not. A time effect was evident on the HVLT-R total and delayed recall scores. All differences between patient and healthy control neuropsychological test scores at baseline were large-sized (ranging from 8.2 to 11.6 points respectively, on average), apart from the digit span total score, which was medium-sized (4.4 and 6.0 points, on average). In every case, patients’ neuropsychological performance was poorer on average, than the healthy controls. Results were similar at follow-up. Nonetheless, both patient and healthy control scores showed improvements on the HVLT-R total recall (patients: Chg = 5.2, *p* < 0.001, Kazis effect size = 0.51; healthy controls: Chg = 3.2, *p* = 0.001, Kazis effect size = 0.49) and the delayed recall (patients: Chg = 3.5, *p* = 0.046, Kazis effect size = 0.33; healthy controls: Chg = 1.7, *p* = 0.16, Kazis effect size = 0.23) scales. Improvements were also noted on the TMT A score (Chg = 3.2, *p* = 0.008, Kazis effect size = 0.51) and digit span total score (Chg = 2.8, *p* = 0.004, Kazis effect size = 0.46) for healthy controls; however, these should be interpreted cautiously, as neither the time effect nor group-by-time interaction were significant.

### Patient-reported outcome measures

#### FACT-Cog subscale scores

Mixed model results are summarized in Table 2. Least squares means for each group at each time, as well as within- and between-groups differences with 95% confidence intervals are summarised in Table 3.

For perceived cognitive impairment and perceived cognitive abilities, fixed effects for time and the group-by-time interaction were statistically significant. Differences between patients’ and healthy controls’ scores on relevant scales at baseline and follow-up were trivial to small-sized (Table 2). Patients’ perceived cognitive impairment was worse at follow-up compared with baseline (− 4.0, *p* = 0.004, Kazis effect size = 0.39), whereas healthy controls was relatively stable (0.4, *p* = 0.63, Kazis effect size = 0.06). Similarly, patients’ perceived cognitive abilities were worse at follow-up compared with baseline (− 2.4, *p* = 0.017, Kazis effect size = 0.43), whereas healthy controls was relatively stable (0.3, *p* = 0.65, Kazis effect size = 0.08). For the impact of perceived cognitive impairment on quality of life, only the fixed effect for group was statistically significant. Differences between patients’ and healthy controls’ scores on the impact of PCI on QOL at baseline and follow-up were large-sized (Table 3), whereas both groups scores were relatively stable over time (patients: Chg = 0.4, *p* = 0.47, Kazis effect size = 0.14; healthy controls: Chg = 0.0, *p* = 0.90, Kazis effect size = 0.02).

#### FACT-G subscale and total scores

Descriptive statistics for the FACT-G, along with estimates relevant to comparisons with general population norms, are provided in Table 4. On average, patients’ physical and emotional wellbeing was worse compared with the general population before (*p* = 0.002 and *p* < 0.001, respectively; medium-sized differences) and after chemotherapy (*p* < 0.001 and *p* = 0.018, respectively; medium-sized differences), but social wellbeing was better (both *p* < 0.001; medium-sized differences). Differences for functional wellbeing and total scores both before and after chemotherapy were trivial to small-sized (all *p* > 0.05).

**Table 4:** Self-reported wellbeing and emotional distress in patients at baseline and follow-up compared with population norms

Measure/(sub)scale	Before chemotherapy					After chemotherapy				
	*n*	*M*	95% CI	Diff	*p*-value	*n*	*M*	95% CI	Diff	*p*-value
FACT-G^a^										
Physical wellbeing	29	21.2	18.8, 23.6	− 3.9	0.002	29	20.6	18.5, 22.6	− 4.5	< 0.001
Social wellbeing	29	23.2	21.3, 25.1	4.0	< 0.001	28	23.4	21.4, 25.5	4.2	< 0.001
Emotional wellbeing	28	16.4	14.5, 18.4	− 4.8	< 0.001	29	19.3	17.9, 20.8	− 1.9	0.018
Functional wellbeing	28	19.5	17.1, 22.0	− 0.8	0.52	29	18.1	15.9, 20.3	− 2.2	0.054
Total score	28	80.2	74.1, 86.3	− 5.7	0.064	28	81.4	75.5, 87.3	− 4.5	0.13
PROMIS emotional distress^b^										
Anxiety 7a	28	55.8	52.0, 59.7	5.8	0.004	27	48.2	44.5, 51.9	− 1.8	0.33
Depression 8b	28	51.5	48.3, 54.7	1.5	0.34	27	49.0	45.4, 52.6	− 1.0	0.57

*Diff* difference between patients’ mean scores and population norm values

^a^For all subscales and the total score, higher scores represent higher wellbeing. General population norm values for the FACT-G (overall): physical wellbeing (*M* = 25.1; possible score range: 0–28), social wellbeing (*M* = 19.2; possible score range: 0–28), emotional wellbeing (*M* = 21.2; possible score range: 0–24), functional wellbeing (*M* = 20.3; possible score range: 0–28), total score (*M* = 85.9; possible score range: 0–108) (Janda et al. 2009). King et al. 2010 evidence-based guidelines for the interpretation of cross-sectional differences: physical wellbeing (1.9, small; 4.1, medium; 8.7, large), social wellbeing (0.7, small; 0.8, medium; -, large), emotional wellbeing (1.0, small; 1.9, medium; -, large), functional wellbeing (2.0, small; 3.8, medium; 8.8, large), total score (6.0, small; 11.0, medium; 22.0, large)

^b^For both short-forms, higher scores represent higher levels of symptomatology and the general population norm value is 50

#### PROMIS Emotional Distress-Anxiety 7a and -Depression 8b

Descriptive statistics for the PROMIS short-forms and comparisons with general population norms are provided in Table 4. On average, patients’ anxious symptomatology was worse compared with the general population before (*p* = 0.004) but not after chemotherapy (*p* = 0.33). Evidence of differences in depressive symptomatology both before and after chemotherapy was weak (*p* = 0.34 and *p* = 0.57, respectively).

### Associations between neuropsychological performance and self-reported cognitive function and emotional distress

Associations between patients’ GDS and FACT-Cog subscale and PROMIS Emotional Distress short-form scores are summarised in Table 5. All associations were trivial or small-sized (all *p* > 0.10).

**Table 5:** Associations between neuropsychological performance and self-reported cognitive function and emotional distress in patients

Measure/scale or short-form	Before chemotherapy		After chemotherapy	
	Kendall’s tau	*p*-value	Kendall’s tau	*p*-value
FACT-cognitive function				
Perceived cognitive impairment	− 0.003	0.99	− 0.06	0.67
Impact of perceived impairment on QOL	− 0.13	0.34	0.09	0.54
Perceived cognitive abilities	0.01	0.94	− 0.15	0.29
PROMIS emotional distress				
Anxiety 7a	− 0.08	0.56	− 0.02	0.90
Depression 8b	− 0.07	0.64	− 0.03	0.85

## Discussion

This secondary analysis explored longitudinal changes in cognitive functioning, wellbeing, and emotional distress in people with newly diagnosed aggressive lymphoma. Cognitive functioning of patients and healthy controls were compared, and wellbeing and emotional distress scores were compared with population norms. Comparisons with population norms and the scores of healthy controls suggest that cancer and cancer diagnosis may impact cognitive function, wellbeing, and emotional distress before commencement of treatment in people with aggressive lymphoma. Findings from our study provide evidence of impaired objective cognitive function in people with newly diagnosed aggressive lymphoma both before and 6–8 weeks after chemotherapy. The cognitive domains affected included attention/working memory, learning memory, speed of information processing, and executive functions as assessed by neuropsychological tests. Compared to a healthy control group well matched in terms of age, sex, marital status, and years of formal education, all differences between patient and healthy control neuropsychological test scores at baseline were large-sized. In every case, patients’ neuropsychological performance was worse, on average, than the healthy controls and remained stable at follow-up. In a study of 249 patients with lymphoma and 212 controls, from pre- to post-chemotherapy and from pre-chemotherapy to 6-month follow-up, patients reported more cognitive problems over time compared with controls and performed statistically significantly worse on tests of verbal memory and delayed recall, attention and executive function, and telephone-based category fluency [6]. A subset of prospective studies in patients with breast, testicular, prostate and colon cancers have confirmed cognitive decline in patients before chemotherapy treatment commences [7, 34–37]. Our findings are consistent with emerging evidence in other cancer populations that cognition is impacted before treatment. The cause of this impairment remains unclear but increasing evidence suggests a direct cancer effect likely via inflammatory pathways, although this hypothesis remains speculative [4]. Therefore, we recommend that “cancer-related cognitive impairment” rather than “chemo-brain” alone should be a focus of research. Screening for cognitive symptoms with clinical intervention during and after treatment for cancer is critical, to ensure patients access adequate support [38]. Preparing people diagnosed with aggressive lymphoma for the possibility of cognitive changes and simple strategies to manage would be the first step in a stepped care pathway to normalise cognitive changes and potentially reduce accompanying emotional distress [39, 40]. To date, it is important to note the aetiology of CRCI remains unclear and hypothesized mechanisms are not well understood and require further evaluation. Further studies evaluating cognitive rehabilitation programs are needed to help patients cope with cognitive difficulties and improve QoL during and after cancer. Future research is needed to better understand this complex problem.

Differences in subjective cognitive function between patients and healthy controls were reflected in self-reported differences on the impact of perceived cognitive impairment on quality of life both before and after chemotherapy. People with newly diagnosed aggressive lymphoma also reported poorer perceived cognitive ability and greater perceived cognitive impairment after chemotherapy. The overall trajectory of cognitive complaints assessed by the FACT-Cog is similar to the pattern of those in Janelsins studies which included lymphoma [6] and breast cancer [41] and other breast cancer studies [42]. Our data show that the diagnosis of aggressive lymphoma is associated with substantial rates of subjective cognitive impairment and patients with a new diagnosis of cancer should be screened for and advised about possible cognitive effects of their disease.

While medium-sized differences in emotional wellbeing were observed between patients and population norms both before and after chemotherapy, there was a reduction in magnitude of the difference. A similar pattern was observed in self-reported anxious symptomatology; on average, the scores of people with newly diagnosed aggressive lymphoma were poorer when compared with population norms before but not after chemotherapy. On average, patients reported higher levels of anxious symptomatology than healthy controls before treatment. The average score exceeded the threshold for mild problems, [43] which is understandable given the diagnosis of a potentially life-threatening illness, uncertainty of prognosis, and commencement of treatment.

Consistent with much of the literature, weak, statistically non-significant associations were observed between patients’ neuropsychological test performance and self-reported cognitive function and emotional distress both before and after chemotherapy [1, 44, 45]. There are several possible explanations for these findings. The first relates to the ecological validity of neuropsychological tests; in this case, the moderate association between neuropsychological test results and the performance of everyday tasks in real world settings [46]. Second, subjective (i.e., self-report) and objective measures of cognitive function likely measure different constructs [7]. It is also possible the neuropsychological tests used in this study did not index aspects of cognition affected by cancer and its treatment. Finally, patients may have been high functioning before their diagnosis and systemic treatment and, while their cognitive function may have declined, it remained within normal limits. Furthermore, self-reported cognitive symptoms have been found to be more strongly associated with other patient-reported outcomes (e.g., mood and fatigue) than with objectively assessed cognitive function [7]. These are important insights for future studies of CRCI.

This study has several limitations. The lymphoma patient sample size was small, and participants were recruited from a single institution. Only patients who were English speaking were eligible as some study assessments were only available in English. Although the study included assessment of cognitive function both before and after chemotherapy, study assessments were limited to 6–8 weeks after chemotherapy, and longer-term follow-up would be useful. This would increase the capacity to explore and describe patterns of CRCI with repeat assessment long into recovery, which is important given the potential for long-term survivorship in this population.

Strengths of our study include a lymphoma patient sample which comprised similar proportions of males and females. A major strength is the inclusion of the healthy comparator group, enabling prospective longitudinal comparison with the lymphoma population. The inclusion of self-report and objective cognitive assessments and other patient-reported outcomes is another strength. Recruitment to and retention in our longitudinal study was excellent, with 30 of 33 people with newly diagnosed aggressive lymphoma recruited over a 10-month period.

## Conclusion

In many people newly diagnosed with aggressive lymphoma, cognitive impairment and the impact of perceived impairment on quality-of-life precede chemotherapy and remain evident after chemotherapy. There is need for larger-scale longitudinal studies over a longer time period with this population in order to inform the development of targeted interventions to address cognitive impairment and the optimal time in the disease trajectory to deliver them.

## Data Availability

De-identified data supporting the findings of this study are available from the corresponding author upon request.
